# Direct current conduction mechanism in the methyl acrylate–vinyl acetate composite thin films

**DOI:** 10.1038/s41598-023-44413-y

**Published:** 2023-10-26

**Authors:** Md. Saddam Sheikh, A. H. Bhuiyan, Mohammad Jellur Rahman

**Affiliations:** 1 Department of Physics, Bangladesh University of Engineering and Technology, Dhaka, 1000 Bangladesh; 2 University of Information Technology and Sciences, Baridhara, Dhaka, 1212 Bangladesh

**Keywords:** Materials science, Organic molecules in materials science, Polymers

## Abstract

Plasma polymerized (PP) methyl acrylate (MA) and vinyl acetate (VA) composite thin films were deposited onto glass substrate varying MA and VA monomer concentrations. Thickness of the composite polymers is observed to vary on the MA and VA monomer ratios, where MA is found more reactive. The FESEM images of the composite polymers show better surface morphology compared to those of the homopolymers. Appearance of broad absorption bands in the FTIR spectra of polymer indicates the structural changes compared to monomer during polymerization. Thermogravimetric analysis and differential scanning calorimetry indicate that composite films are thermally more stable (up to 617 K) compared to homopolymer thin films (563 K). The current density versus voltage (*J–V*) characteristics of PP(MA-VA) composite films (sandwiched between aluminum electrodes) with different MA and VA ratios showed that the *J* values of the composite films gradually increase with elevating VA monomer and also with temperature (298–373 K). On the other hand, this value increases with decreasing the thickness of the composite films, which complies with the other studies. The conduction of the thickness-dependent composite films showed Ohmic in nature in the lower voltage region (< 10 V) while the space charge-limited conduction is found to be dominated in the higher voltage region (> 10 V) operating over the entire range of temperature. The activation energy at room temperature was found to be ~ 0.019 eV in the Ohmic region and 0.260 eV in the non-Ohmic region.

## Introduction

Researchers have been continuously investigating the optical, structural, thermal and electrical properties of plasma polymerized (PP) organic thin films. These films become increasingly popular owing to their unique characteristics and inclusive range of applications in sensors and coatings, as a tool of surface hardening, sacrificial layers, low-dielectric-constant interlayers in microelectronics and interconnected technologies, numerous spaceship components and optoelectronic devices^1–5^. Electronic waste is a growing problem worldwide. To address this issue, researchers and manufacturers are exploring organic biocompatible and biodegradable polymeric composite materials to invent safe electrical, electronic, or optoelectronic devices to deal with this electronic garbage^6–8^.

Composite materials, in particular, have gained significant attention in current years due to their aptitude to create modified electrical properties with high thermal stability^9,10^. The composition of two or more monomers in the production of polymers may lead to the development of high-strength and lightweight materials for specific applications that are used in a variety of sectors including surface modification, altered electrical, mechanical, and optical properties, metal protection, the design of complicated materials, and so on^11,12^. These demand for materials with improved properties has stimulated the development of new material assortments, alloys, and composites.

In this study, the plasma polymerization technique has been chosen to deposit composite thin films since this process does not necessitate any external reagents, produces no extra waste, and is environmentally friendly. The films gained by this technique are homogeneous and highly resistant to chemical and physical treatments^13^. Moreover, this process seems to be suitable due to its various benefits including low cost, ease of synthesis, and lack of any energy-intensive heating and cooling cycles^14,15^.

The reports on the effect of mixing two or more organic-organic monomers for preparing composite thin films are limited in the literature. However, a few researchers have reported on composite and bilayer films deposited using different techniques. Islam et al.^16^ synthesized methyl methacrylate and vinyl acetate composite polymers by suspension polymerization method and reported that the resulting polymer developed chemical and physical properties compared to homopolymers made from only one of the monomers. The composite polymer was found to be thermally more stable than the homopolymers. PP Composite thin films also reported to have significantly enhanced ionic conductivity with the addition of filler^17^.

A recent publication^18^ reported that poly(pyrrole-co-aniline) composite polymers made of atmospheric pressure plasma polymerization showed higher electrical resistance with a higher deposition rate at a suitable pyrrole ratio (pyrrole at a ratio of 75% to aniline at 25%). The surface morphology and the composite film thickness were also observed to be dependent on the variation of the two monomer mixture ratios because of the rapid rearrangement or incorporation of the monomer units. Additionally, Akter et al.^19^ reported that the electrical conductivity of the PPTMA thin film can be adjusted by controlling the iodine doping concentration on the pure PPTMA film using the plasma polymerization method. The iodine-doped films showed two types of electrical behavior depending on the applied voltage and in the higher voltage the films obey the space charge limited conduction (SCLC) mechanism. Therefore, study of electrical properties of pp thin films could be a good choice of research.

In this study, methyl acrylate (MA) and vinyl acetate (VA) have been elected to prepare the composite films. Because the MA can be copolymerized easily with other monomer molecules to form various copolymers. The produced poly(methyl acrylate) and its copolymers have a wide range of electrical applications owing to their excellent dielectric properties and mechanical strength. VA is chosen as the other monomer because it is widely used in the production of polyvinyl acetate (PVA) and its copolymers. Both of the monomers have excellent dielectric properties and were used in electrical insulation materials, solder resists and conformal coatings to protect the conductive traces on the printed circuit board from oxidation^16,20,21^. Moreover, having a particular electron-donating acetoxy group (–OOCCH_3_) group associated with the vinyl group, VA is considered a less-activated monomer with a lower deposition rate^22^. When a composite is prepared from such two monomers (one more active and the other less active) their optical and structural properties are observed to vary depending on the monomer ratio and thus the electrical properties may also vary. To recognize the electrical conduction processes in the composite films charge generation and transfer in the interfaces and bulk of the films are important to be studied. Several researchers studied the current density versus voltage (*J–V*) characteristic in organic thin films equipped with different methods^19,23–25^. The proper choice of materials include smoothness, porosity level, thermal stability, high voltage capability, and conduction. Deviation of these properties with temperature are critical factors in ensuring that the films could be efficiently utilized in electrical and electronic applications. However, the study on composite films to improve the material system and to understand how conduction operates in composite thin films is very rare. Thus, it is crucial to gain a thorough insight into the carrier transport mechanisms and how it function in the PP(MA-VA) composite thin film along with other properties.

In this work, electrical and thermal properties of the PPMA and PPVA homopolymer and PP(MA-VA) composite polymer thin films with surface morphological and structural properties were methodically examined using different ratios of MA and VA. The thermal stability and temperature-dependent DC electrical properties besides the conduction mechanism of the thickness-dependent composite films have been studied to explore the potential compatibility of the thin films for electrical and electronic devices of modified required applications.

## Materials and methods

### The monomers

**Figure 1:**
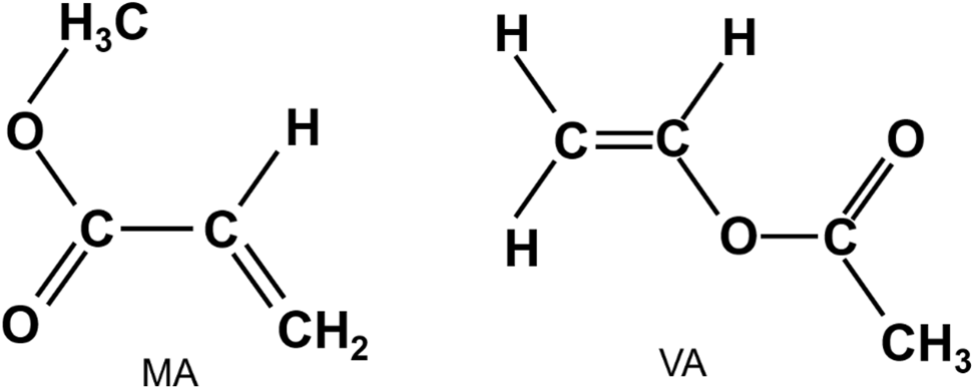
Chemical structure of the methyl acrylate and vinyl acetate.

MA and VA monomer precursors used for preparing the PP composite thin films are obtained from BDH, Poole, England and Merck, Germany, respectively. The chemical formula, boiling point and density of MA are CH_2_CHCO_2_CH_3_, 353 K and 950 kg/m^3^ and those of VA are CH_3_CO_2_CHCH_2_, 345.7 K and 934 kg/m^3^, respectively. However, the molecular weight of both monomers is same (86.09 g/mol) and the chemical structures of the monomers are shown in Fig. 1

### Experimental details

The pre-cleaned glass slide (25.4 $\times$ 76.2 $\times$ 1.2 mm^3^, Sail brand, China) are taken to deposit PP thin films. A detailed explanation of the plasma polymerization process is described in earlier research^26^. The plasma reactor consists of two electrodes made of stainless steel that are placed in close proximity to each other to create a uniform electric field. Before starting deposition the plasma chamber is vauumed to a low pressure of 0.1 Torr using a rotary pump. To ignight discharge plasma glow the electrodes are connected to an AC (50 Hz, 22 W) power supply and the films are deposited for 60 min. The vapor of the composite monomer solution entered into the plasma chamber due to highly pressure difference between the monomer container and the chamber. The flow rate is kept constant at ~ 20 cm^3^/min with the help of an injecting valve. The films are deposited by fluctuating the amount of MA (100%, 75%, 50%, 25% and 0%) in the mixture of MA and VA monomer and the films are defined as PPMA, PP(MA-VA) (3:1), PP(MA-VA) (1:1), PP(MA-VA) (1:3) and PPVA respectively.

**Figure 2:**
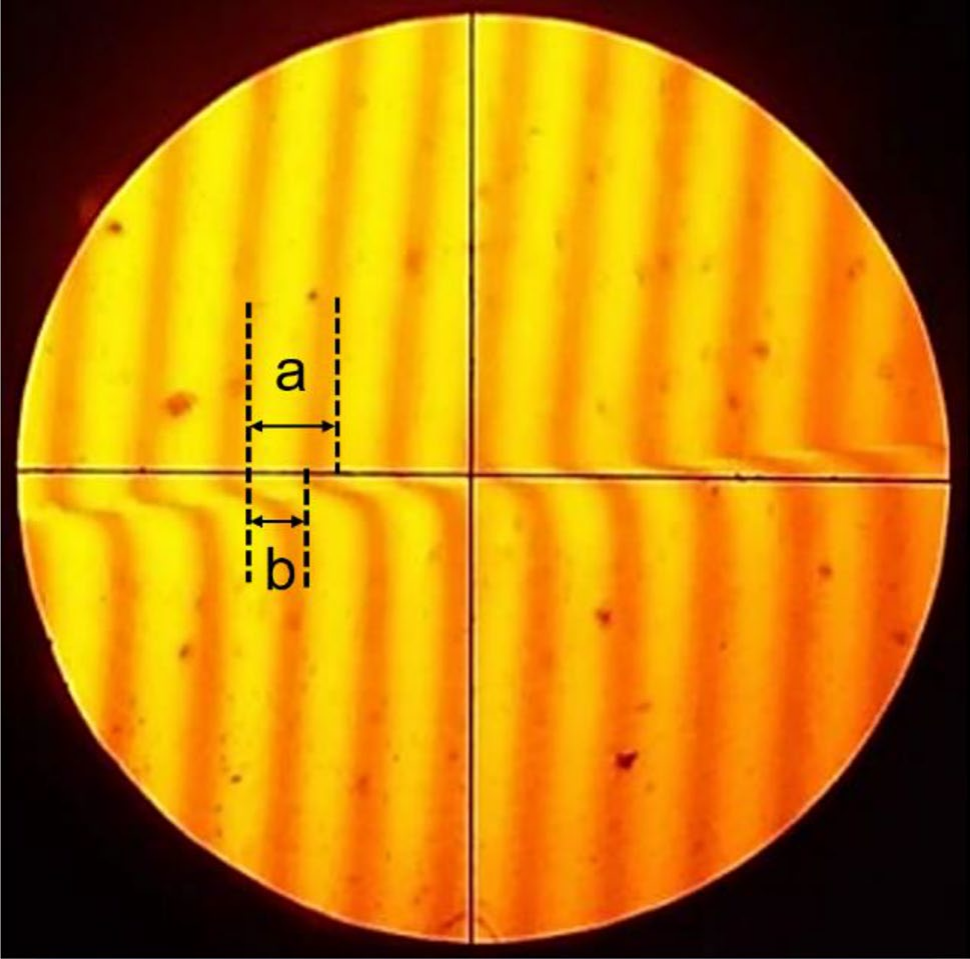
Image of the fringes occupied from multiple-beam interferometric set-up.

The as-deposited film thicknesses, *d*, are measured by multiple-beam interferometric technique^27^ using the following equation:

1 $$d =\frac{\lambda }{2}\frac{b}{a}$$

where *λ* (= 589.3 nm) is the wavelength of the light source (sodium) and $\frac{b}{a}$ is the fractional discontinuity in the produced fringe interfaces as shown in Fig. 2. Before deposition half of the substrate was covered using Teflon taps and the remaing uncovered side was allotted to deposit the films. The film’s thickness was measured at multiple points, and the average thickness was calculated based on at least 8 measurements. All of the films are deposited in the same plasma conditions (for 60 min) but the thicknesses of the resulting films are found to be 213 ± 7, 197 ± 4, 175 ± 4, 137 ± 5 and 117 ± 4 nm for PPMA, PP(MA-VA) (3:1), PP(MA-VA) (1:1), PP(MA-VA) (1:3) and PPVA, respectively. This is because MA is more reactive monomer compared to VA. Although both MA and VA monomers contain a vinyl group (CH_2_=CH–), there are certain structural differences that can influence their reactivity. MA has an ester group (–COOCH_3_) adjacent to the vinyl group and the presence of the ester group introduces an electron-withdrawing carbonyl functionality^22^. This increased reactivity of MA lead to faster and more efficient polymerization reactions to form plasma polymerized films during the polymerization process. In contrast, VA contains an additional electron-donating acetoxy group (–OOCCH_3_) and this nature can stabilize the electron density around the vinyl group, making it less reactive toward the nucleophilic attraction. As a result more deposition rate of MA increase the film's thickness even the plasma is operated for the same duration.

### Characterization techniques

Surface morphology of the as-deposited thin films is examined at an accelerating voltage of 5 kV using field emission scanning electron microscope (FESEM) (JEOL JSM 7600F, USA). The deposited films are masked with platinum to evade charging effects throughout the measurement.

The associated functional groups of the films are observed by a double-beam Fourier transform infrared spectroscopy (FTIR) (SIMADZU, FTIR-8400) with a resolution of 2 cm^−1^ to elucidate the chemical structure of the homo polymer and composite polymer thin films.

To observe thermal stability, thermogravimetric (TG) and differential scanning calorimetry (DSC) are done under N_2_ atmosphere using a computer-controlled TG/DSC thermal analyzer (Model: NETZSCH STA 449 F3 Jupiter). The heating rate was kept at 10 K/min and the analyzer employs a horizontal system balance mechanism. The powder samples for FTIR spectroscopy are gathered from the substrate’s surface.

### DC electrical measurement

**Figure 3:**
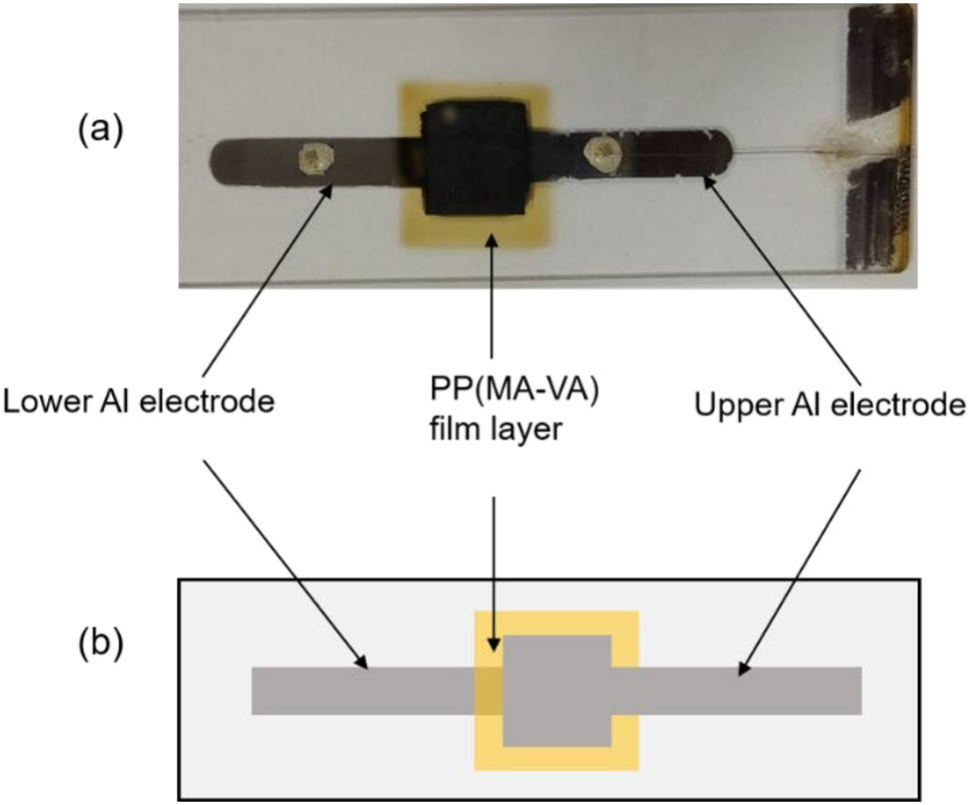
(**a**) Photograph of the electrical sample and (**b**) schematic drawing with electrode assemblage of Al/PP(MA-VA)/Al sandwich-type thin film.

Pure (99.99%) aluminum (Al) (Willacy, Japan) is used for electroding the samples, and Al/PP(MA-VA)/Al sandwich-type structure is prepared for electrical measurements as shown in Fig. 3. Firstly, the lower Al electrode is deposited on the pre-cleaned glass slide using a vacuum coating unit (Model:12A4D, Hind High, India) at a very low pressure (1.33 $\times$ 10^−3^ Pa) and then PP film is deposited on the lower electrode and finally the upper electrode is similarly deposited on the film in the opposite direction. The lower and upper electrodes are prepared in a squared shape mask with an effective area of 10^−4^ m^2^ because the current density depends on the electrode area. Keithley 614 a high-impedance electrometer is used to monitor current flow and Agilent 6545A is used as a stabilized DC power supply. The electrical sample is placed into a heating chamber, which is evacuated to ~ 0.2 Torr to eliminate any ambient impact and the electrical measurements are carried out in the temperature range of 298 to 398 K, where the temperature inside the vacuum chamber is recorded by placing a Cr-Al thermocouple in the vicinity of the sample.

## Results and discussions

### Analysis of surface morphology

**Figure 4:**
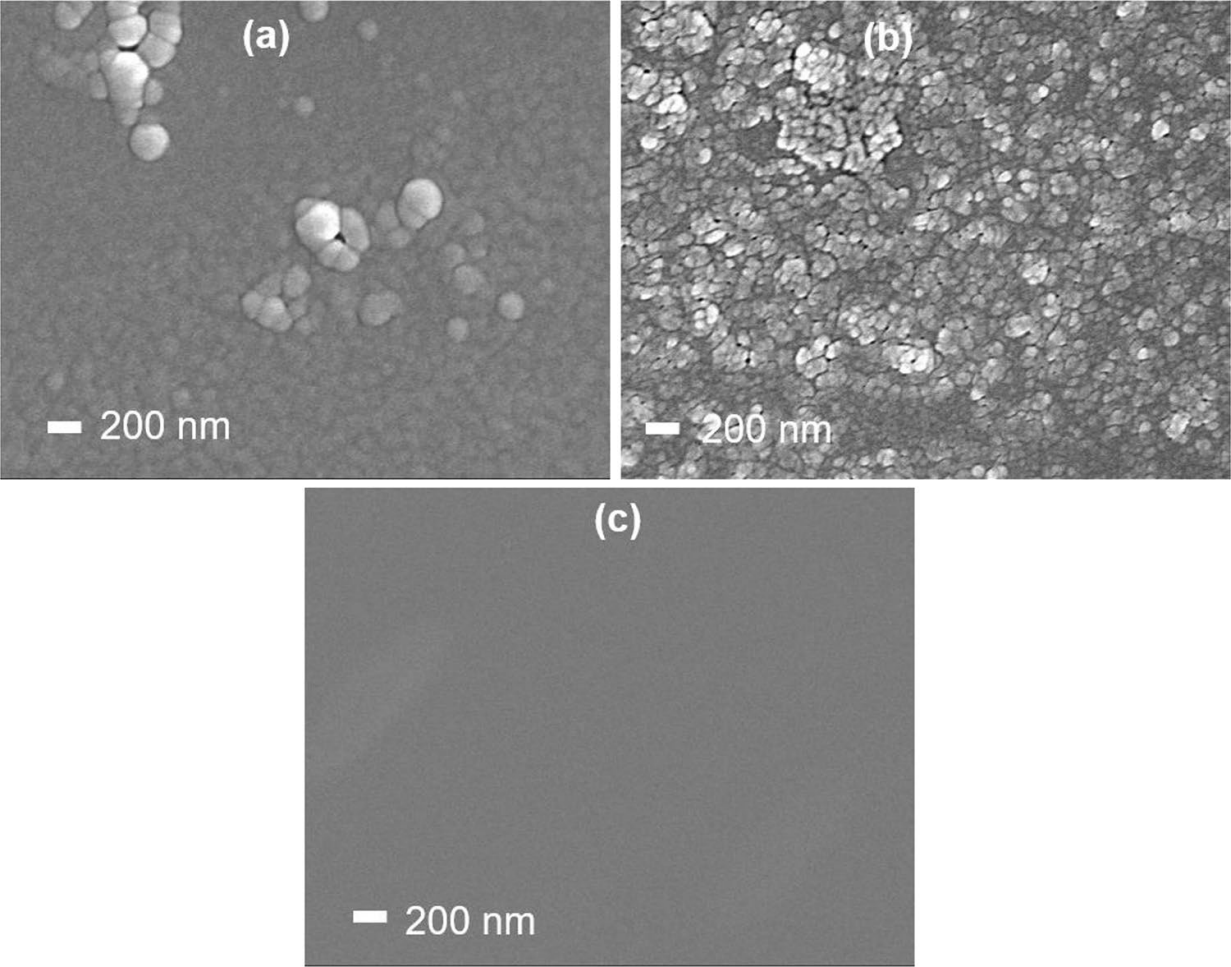
FESEM images of (**a**) PPMA, (**b**) PPVA and (**c**) PP(MA-VA)(1:1) thin films.

Figure 4 depicts the FESEM images of homo polymer and composite polymer thin films captured at a magnification of 50 k. The PP(MA-VA)(1:1) composite film is observed to be more smooth, scratch-less, and pinhole-free compared to the PPMA and PPVA films surface. No visible grain or cluster is found on the composite film surfaces, while some cluster or mosaic-type structures are visible on the homo polymer surfaces. This type of mosaic-like surface was also found for homopolymers in some other studies deposited by plasma polymerization technique^28,29^. It is speculated that the conformation of two distinct monomers boosted the crosslinking among the plasma radicals, ions, or molecules by the collisions of energetic electrons, which reduces the polymer chain and resulted in improved surface morphology of the composite films^30^. A details description of surface morphology is reported in our earlier research work^31^.

### FTIR analysis

The FTIR spectra of the homo polymer and composite polymer films were described in detail in our recent publication^31^. The presence of absorption peaks related to the stretching vibration of methyl group (–CH_3_) and the bending vibration of C–H at 2922–2970 cm^−1^ and 1378 cm^−1^^32^, respectively, confirmed the organic nature of the compounds. In the fingerprint region of the MA and VA monomers, some sharp absorption peaks appear but all the peaks are not present or merged in the spectra of the deposited films. This indicates the reorganization or recombination of the monomer molecules and formed highly crosslinking PP thin films^32^.

### Thermal analysis

**Figure 5:**
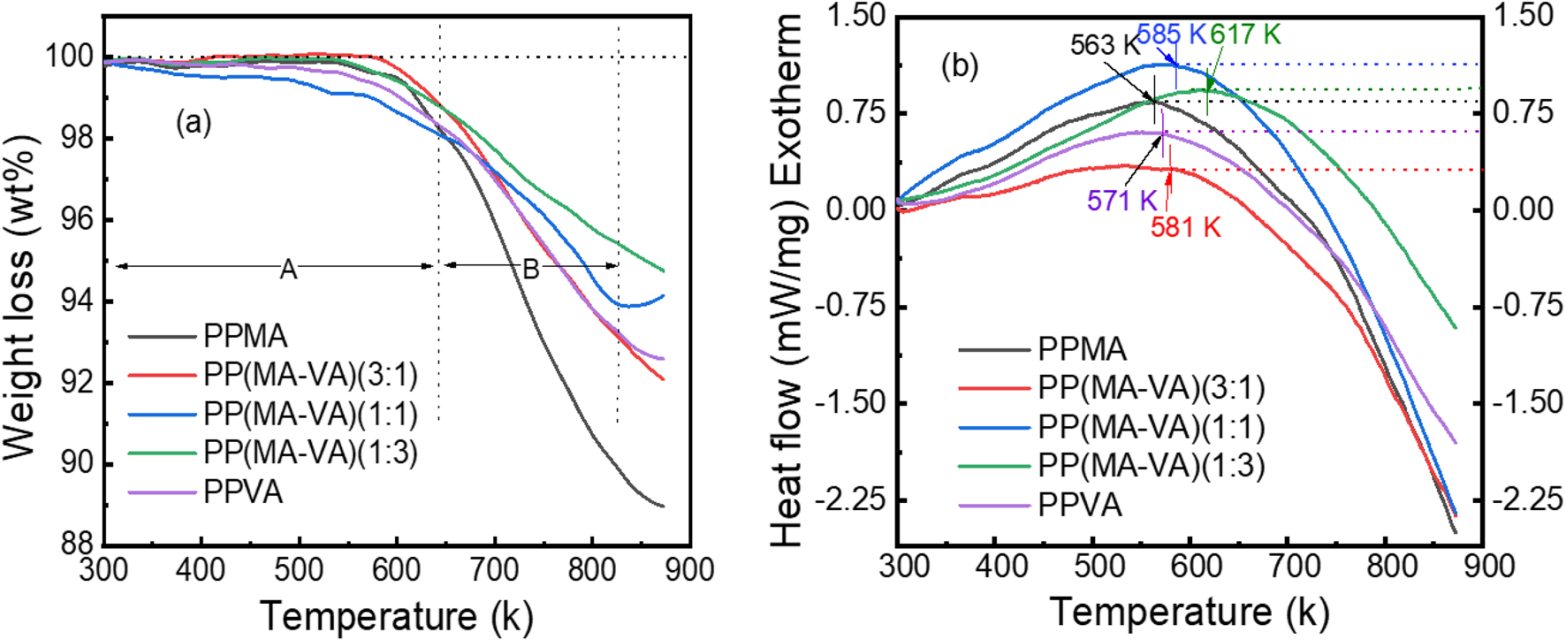
(**a**) TG, and (**b**) DSC graphs of the as-deposited PPMA, PPVA homopolymer and PP(MA-VA) composite polymer thin films.

The TG curves of PPMA, PPVA, and PP(MA-VA) powder samples taken under N_2_ environment are shown in Fig. 5a and provide valuable information about thermal stability of the thin films. TG is a technique that tracks the percentage of weight variation of a sample during a heating process, and the resulting curves provide information on the thermal degradation of the samples. In this case, the TG curves show two stages of degradation, A and B, with the gradual increase in temperature. At temperatures between 303 to 642 K (region A) minimal weight loss (1.2–1.4%) is observed, which may owing to the loss of low molecular mass species like H_2_O and CO_2_, which are generated during the thermal stabilization process^33^. This low percentage of weight loss in these regions also indicates the hydrophobic behavior of the as-deposited PP thin films.

On the other hand, region B shows significant weight loss due to the thermal decomposition or breakdown in the polymer chain. The percentage of thermal decomposition is 7% and 10% for PPVA and PPMA homopolymers, respectively, and approximately 5%, 6%, and 7% are for the PP(MA-VA) (1:3), PP(MA-VA)(1:1) and PP(MA-VA)(3:1) composite polymer films, respectively. However, the weight loss decreases with the increased amount of VA in the PP(MA-VA) composite films. This is due to the development of stronger crosslinking during the formation of composite polymeric chains, which increases the thermal stability of the films and helps the films to resist the molecules being thermally degraded^34^.

The DSC melting curves of homo polymers and composite polymers as shown in Fig. 5b, exhibit significant degradation of exothermic peaks. The maximum stability temperatures of PPMA and PPVA are 563 K and 571 K, respectively. Beyond this temperature, an endothermic reaction is initiated due to the thermal breakdown of the polymering chains. However, for the composite polymer samples of PP(MA-VA) (1:3), PP(MA-VA)(1:1) and PP(MA-VA)(3:1), the exothermic peaks are observed at around 617, 585, and 581 K, respectively. As the VA monomer concentration increases, the exothermic band peaks shift towards a higher temperature range. This suggests that PP(MA-VA) composite films are more thermally stable than any of the homopolymers due to the copolymerization effect.

### The current density versus voltage characteristics

**Figure 6:**
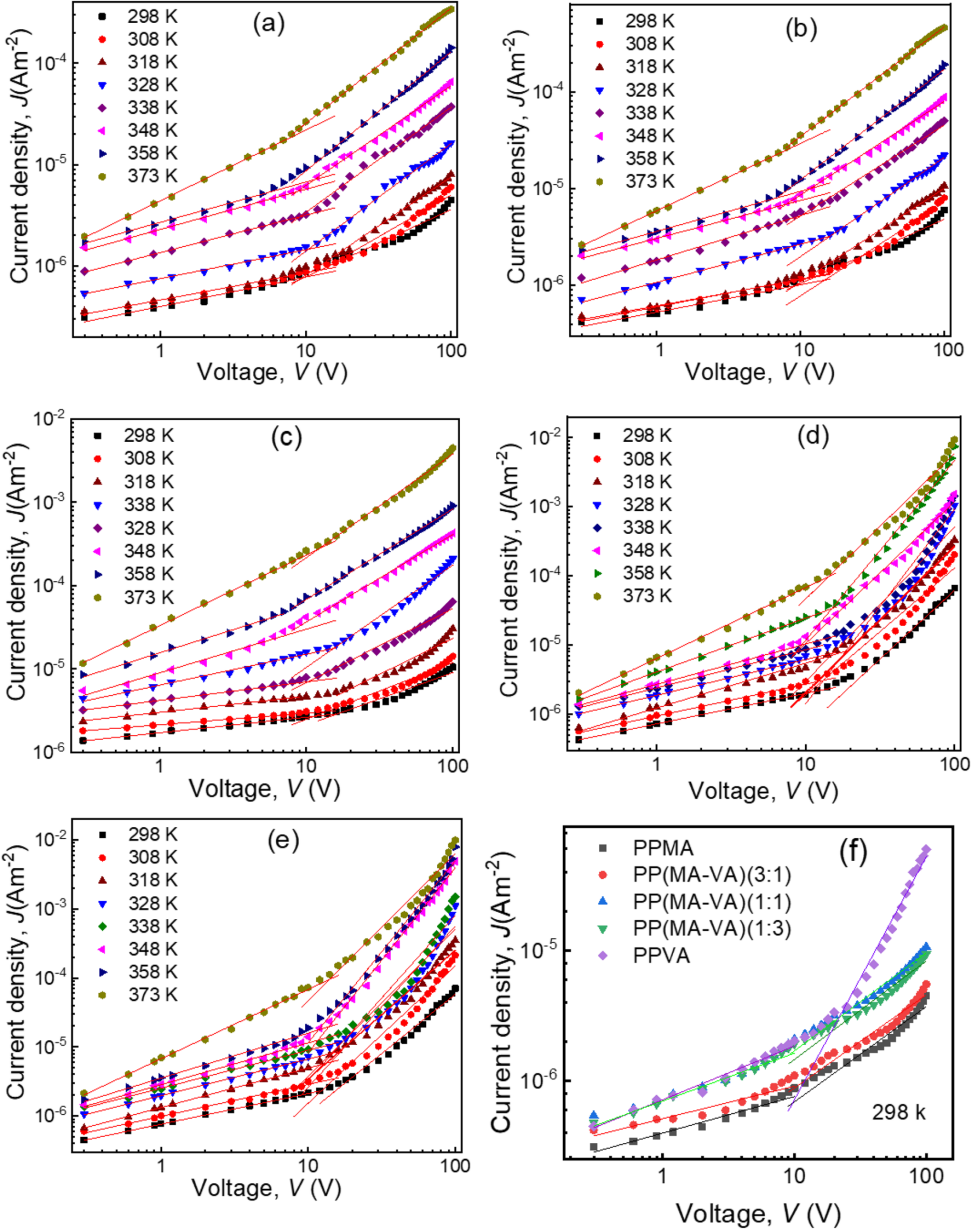
*J–V* characteristics of (**a**) PPMA, (**b**) PP(MA-VA)(3:1), (**c**) PP(MA-VA)(1:1), (**d**) PP(MA-VA)(1:3), (**e**) PPVA and (**f**) at room temperature of the composite thin films of different thicknesses.

The *J–V* characteristics of PPMA and PPVA homo polymer and PP(MA-VA) composite polymer thin films are recorded at various temperatures within the voltage range of 0.3 V to 100 V and the temperature range of 298 K to 373 K. Figure 6 indicates a change in the current density in the same voltage region for all homo polymer and composite polymer thin films. Moreover, it can be observed that *J* increases as the proportion of VA monomer in the composite structures increases. This may be due to decline the thickness of the composite film with VA monomer, resulting in more electron transfers from the highest occupied molecular orbital (HOMO) to the lowest unoccupied molecular orbital (LUMO) level and the creation of traps that enhance the electrical conductivity^35^. In polymers, these traps are considered as extrinsic charge carriers.

The *J–V* characteristics of the homo polymer and composite polymer films obey a power law of *J* ∝ *V*^*n*^, where *n* is the power index, and the slopes of *n* ≤ 1 indicate an approximately Ohmic type conduction, while 1 < *n* represents a non-Ohmic conduction at higher voltages^23,36^. The *n* value of all the as-deposited films are found to lie in the range of 0.15 to 0.98 in the low voltage region (≤ 10 V) and 1.00 to 2.65 in the high voltage region (> 10 V), respectively indicating Ohmic conduction and non-Ohmic conduction mechanism, respectively.

### Thickness-dependent conduction mechanism

**Figure 7:**
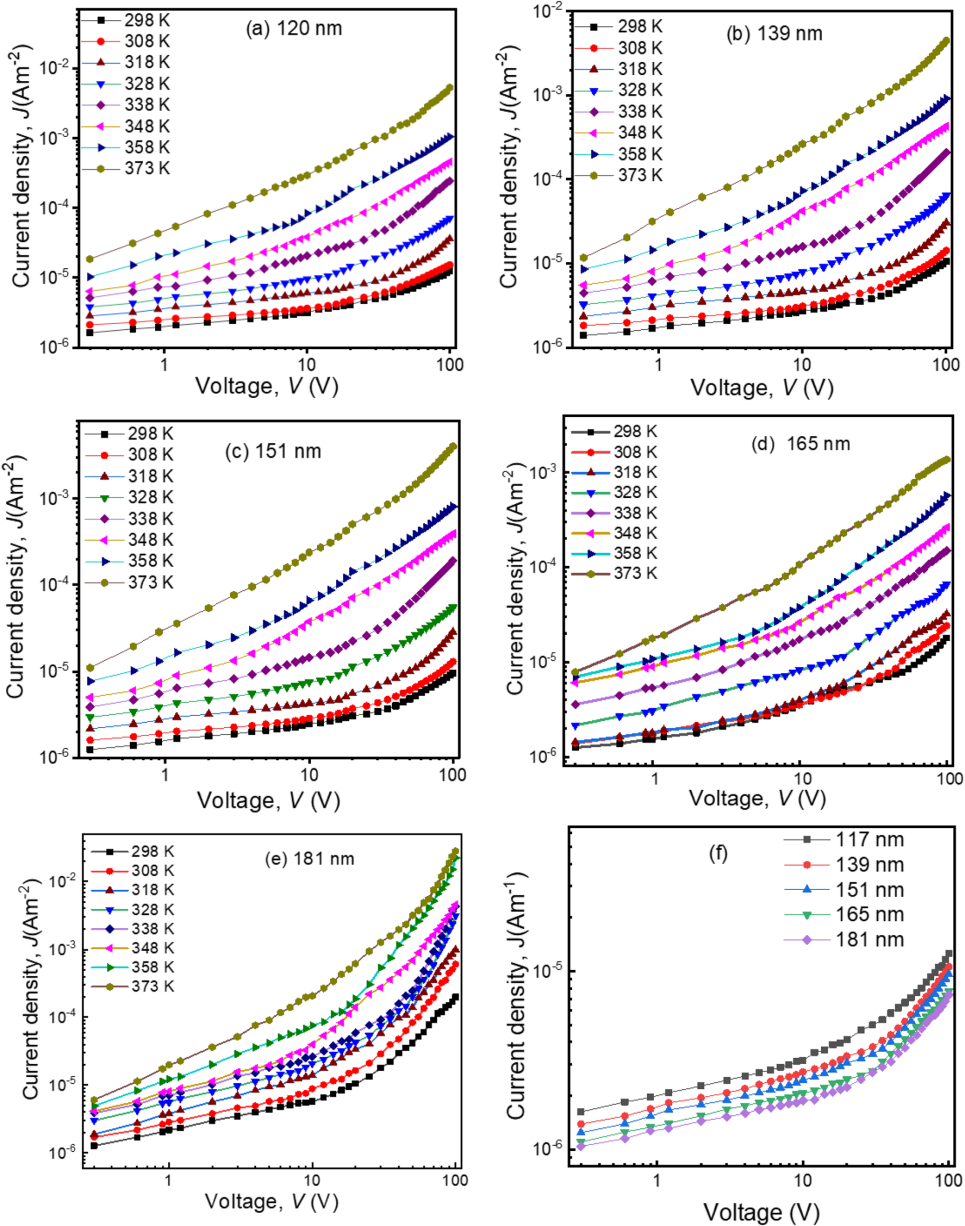
*J–V* relationship of the PP(MA-VA) composite thin films of thicknesses (**a**) 120 nm, (**b**) 135 nm, and (**c**) 165 nm at different temperatures, (**d**) for the different thi cknesses at room temperature (298 K).

To determine the direct current (DC) conduction mechanism in the thickness-dependent composite thin films, the PP(MA-VA)(1:1) is deposited for 30, 45, 60, 75, and 90 min and the thicknesses of the films are observed to be 117 ± 5, 139 ± 6, 151 ± 5, 165 ± 4 and 181 ± 6 nm, respectively. The *J–V* characteristics curves of these films are shown in Fig. 7, and it is observed that the *J* increases as the film thickness decreases within the same applied voltage. This may be attributed to a decrease in the number of grain boundaries as the film thickness decreases, which in turn reduces the barriers to charge transport and increases conductivity. Additionally, a decrease in film thickness reduces the distance between the electrodes leading to an increase in electric field strength, which also contributes to an increase in current density^37^. These findings are consistent with some previous reports^23,38^.

**Figure 8:**
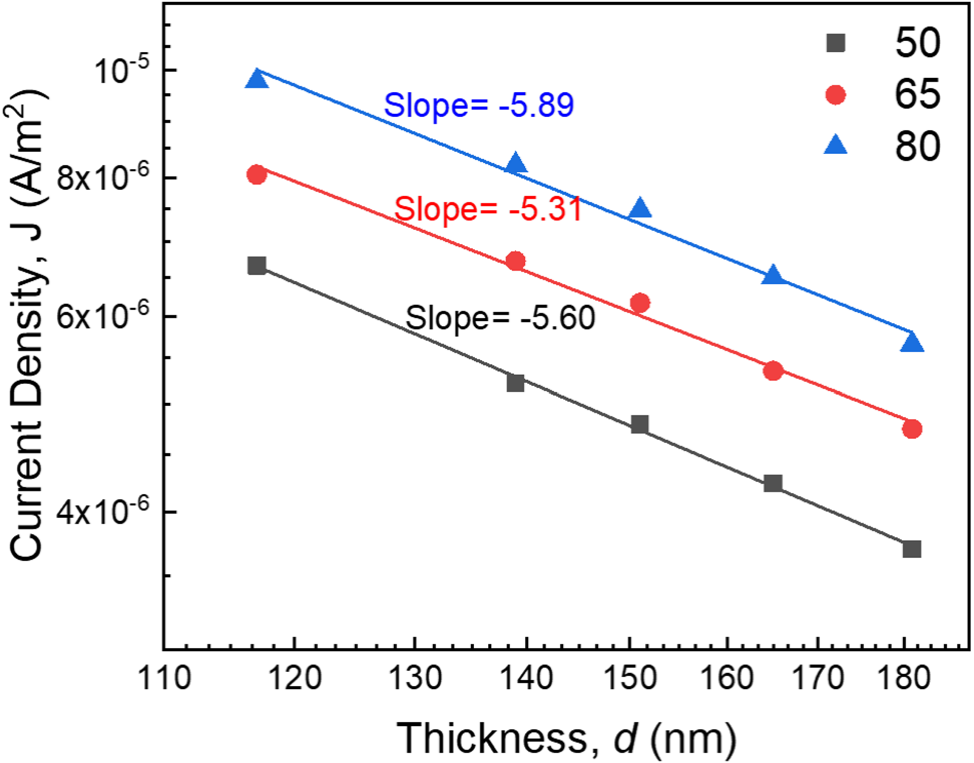
Plots of *J* versus *d* for PP(MA-VA) composite thin films in the non-Ohmic region (at 50, 65 and 80 V).

In the lower voltage region (< 10 V), the value of *n* is observed between 0.16 and 0.98 for the thickness dependent samples again indicating an Ohmic-type conduction mechanism. At higher voltages, the *n* value is between 1.00 to 2.65, suggesting a non-Ohmic conduction mechanism, which may be space charge limited (SCLC), Schottky, or Poole–Frenkel (PF)^36,39^. The dependence of *J* on the distance between the electrodes, that is the thickness of the films (*d*) follows the power law *J* ∝ *d*^*−l*^, where *l* is a parameter that depends on the presence of traps in the films. When |*l*|≥ 3 the mechanism would be SCLC and when |*l*|< 3 the mechanism would be Schottky or PF^36,40^. Therefore, to investigate the conduction mechanism in the PP(MA-VA) films, *J* is plotted against *d* as shown in Fig. 8 for the thicknesses dependent films in the non-Ohmic region at three different voltages of 50, 65 and 80 V. In all the cases the negative slopes are 5.89, 5.31 and 5.60, respectively, indicating that neither Schottky nor PF mechanisms are present in these films. The main conduction mechanism in these films is SCLC. In SCLC process, when an external voltage is applied across the film, charge carriers (electrons or holes) are introduced from electrode to the polymer. These carriers then interact with traps, which are defects or localized states with energy variations within the band gap of the material. In these circumstances, the carriers might get stuck into these traps or move through the material maintaining dynamic equilibrium^41,42^. With an increase in voltage, more carriers are injected, causing a buildup of charges close to the injection electrode. This accumulation generates an internal electric field that counteracts further carrier injection, leading to the distinctive non-Ohmic behavior characteristic of SCLC^41,42^.

### Dependence of J on temperature

In a polymer system, carrier mobility is usually deficient. Temperature, concentration of conducting species, dopant level, polymer shape and other parameters can affect the conductivity. With temperature, the charge carrier density grows sharply, which indicates the temperature dependence on conductivity, but poor field conduction results in an exponential temperature dependency. The Arrhenius equation (Eq. 2) is commonly used to analyze the effect of temperature on current density^43^.

2 $$J={J}_{0}\mathrm{exp}\left(-\frac{\Delta E }{{k}_{B}T}\right)$$

**Figure 9:**
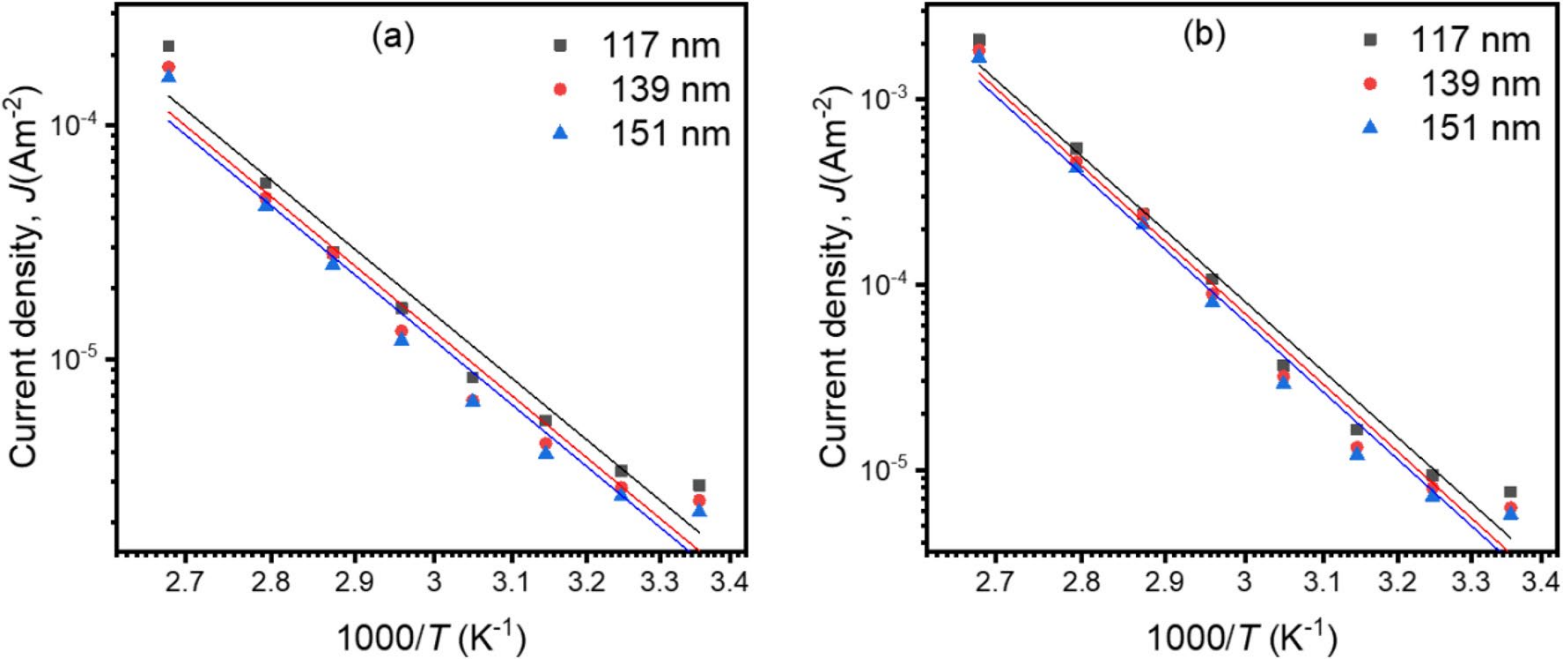
Plots of* J* versus *1/T* of thickness-dependent composite thin films in the (**a**) Ohmic and (**b**) non-Ohmic regions.

Where, *k*_*B*_ = Boltzmann constant, *ΔE* = Activation energy and *J*_0_ = Current density at thermal equilibrium. The dependence of *J* on 1/*T* for thickness-dependent composite films is shown in Fig. 9. The curves indicate that the conductivity is elevated with higher temperature in both Ohmic and non-Ohmic regions, which leads to the higher *ΔE* with temperature. The values of *ΔE* are almost 0.019 eV in the Ohmic region (at 7 V), and 0.260 eV in the non-Ohmic region as presented in Table 1. These changes in *ΔE* are related to the structural changes of the polymer with temperature.

**Table 1:** Values of activation energy *ΔE* (eV) for thickness-dependent PP(MA-VA) at Ohmic and non-Ohmic voltage regions.

Thickness, *d* (nm)	Activation energies, *ΔE* (eV)	
	Ohmic	Non-Ohmic
120	0.015	0.201
137	0.015	0.202
165	0.019	0.260

In polymer, the charge carrier moves by transferring the thermally triggered charge carriers among the localized states over a potential barrier, which is known as hopping conduction^44^. To overcome the potential barrier, those charge carriers obtain adequate energy from the lattice through thermal oscillations. The reduction in *ΔE* with declining temperature indicates a successive transition to the hopping regime. Therefore it is inferred that trap-mediated SCLC mechanism is observed in this thickness-dependent composite thin films.

## Conclusions

A low-pressure capacitively coupled glow discharge reactor is used to successfully deposit desired PPMA, PPVA, and PP(MA-VA) films onto glass substrates. The combination of two organic monomers increased the polymerization rates and allowed a wider range of polymeric properties. The thickness of the PP(MA-VA) composite films decreased from 213 to 117 nm as the concentration of MA monomer in the mixture decreased. Composite polymer films showed improved surface morphology with smooth, homogeneous, and pinhole-free surfaces as compared to PPMA and PPVA homopolymers. During plasma polymerization the structure of the monomer changes significantly due to the reorganization of molecules. The composite films are thermally stable up to 617 K, and this stability increased with a higher concentration of VA. The electrical conductivity increased for the sample of lower thickness and at higher temperatures. The maximum value of *ΔE* at room temperature in the Ohmic region is 0.019 eV, and this value is elevated to 0.260 eV in the non-Ohmic region at 60 V. The dominant electrical conduction mechanism in the PP(MA-VA) thickness-dependent composite thin films is SCLC in the non-Ohmic region. This study concludes that the composition of MA and VA monomers significantly improves the structural, thermal, and electrical properties of the composite films, making them suitable for various electronics and optoelectronics devices.

## Data Availability

The datasets used and/or analyzed during the current study are available from the corresponding author upon reasonable request.
